# Characterization of AmtA, an amidinotransferase involved in the biosynthesis of phaseolotoxins

**DOI:** 10.1002/2211-5463.12071

**Published:** 2016-05-16

**Authors:** Mi Li, Li Chen, Zixin Deng, Changming Zhao

**Affiliations:** ^1^ Key Laboratory of Combinatorial Biosynthesis and Drug DiscoveryMinistry of EducationSchool of Pharmaceutical SciencesWuhan UniversityChina

**Keywords:** amidinotransferase, biosynthesis, homoarginine, phaseolotoxin

## Abstract

Phaseolotoxins (PHTs), which are produced by *Pseudomonas*, belong to a family of phosphoramidate natural products. Two nonproteinogenic amino acid precursors, N^δ^(N′‐sulfo‐diaminophosphinyl)‐ornithine (PSOrn) and homoarginine (*h*Arg), are involved in biosynthesis of PHTs. Amidinotransferase AmtA catalyses the formation of *h*Arg, with arginine and lysine as substrates. AmtA was overexpressed and purified in an *Escherichia coli* system. An *in vitro* enzyme assay showed that it has stricter substrate specificity than certain other amidinotransferases. Site‐directed mutagenesis experiments showed that the mutation AmtA Met243His244 is an alternative while Met246 is essential for the transamidination activity.

**Abbreviations**
AGAT: l‐arginine:glycine amidinotransferase
hAGAT: Human l‐arginine:glycine amidinotransferase
*h*Arg: homoarginine
l‐Dap: l‐2,3‐Diaminopropionic acid
OTCase: ornithine transcarbamylase
PHT: Phaseolotoxin
PSOrn: N^δ^(N′‐sulfo‐diaminophosphinyl)‐ornithine

Phaseolotoxin [N^δ^(N′‐sulfo‐diaminophosphinyl)‐ornithyl‐alanyl‐homoarginine, PHT] is a tripeptide natural product produced by phytopathogenic bacteria *Pseudomonas syringae* pv. *phaseolicola* and *actinidiae* 1, 2. PHTs contribute to the pathogenicity of pathogens and confer the latter with survival advantages as well 1, 3. N^δ^(N′‐sulfo‐diaminophosphinyl)‐ornithine (PSOrn), the pharmacophore of PHT, could work as an irreversible inhibitor of ornithine transcarbamylase (OTCase) *in planta* 4, 5. Our previous works showed that PHT‐resistant OTCase ArgK plays a dual role for the self‐defense of PHT‐producing *Pseudomonas*. It controlled the production of PHTs in addition to providing an alternative arginine source 6. There are two nonproteinogenic amino acid precursors, PSOrn and homoarginine (*h*Arg), involved in PHTs biosynthesis (Fig. 1). Even though the PHTs biosynthetic gene cluster (*pht*) has been characterized, there is still something unrevealed existing in the biosynthetic pathway for PSOrn 6, 7, 8. The *h*Arg was considered to be formed with the catalysis of amidinotransferase AmtA while arginine and lysine perform as substrates (Fig. 2A) 9, 10. However, the biochemical characteristics of AmtA are poorly understood.

**Figure 1:**
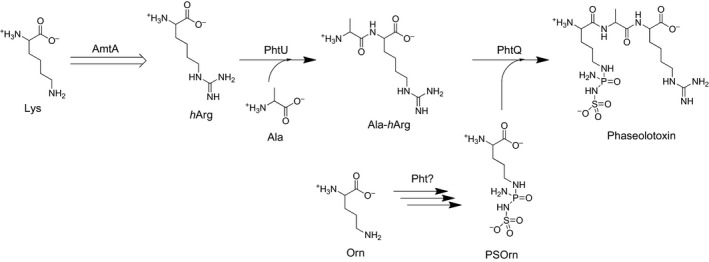
Proposed biosynthetic pathway of phaseolotoxins.

**Figure 2:**
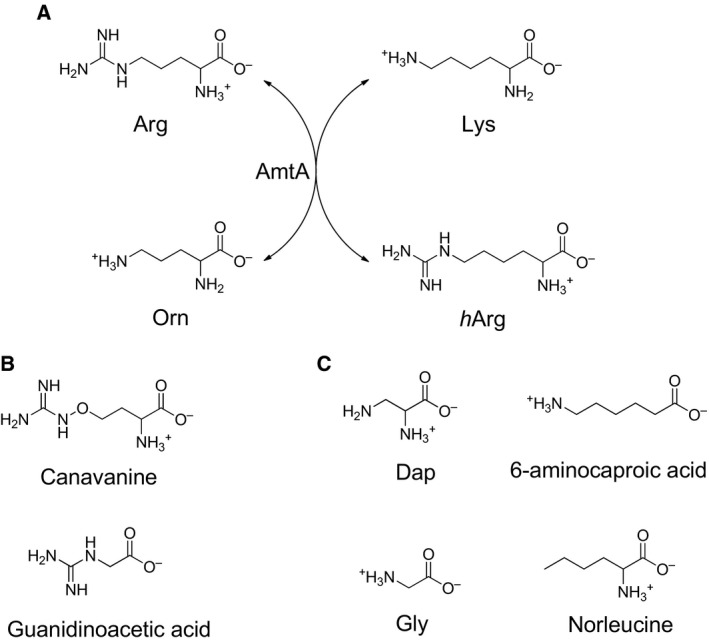
Scheme of transamidination reaction. (A) AmtA catalyzed *h*Arg formation; (B) amidine donors tested in this work; (C) amidine acceptors tested in this work.

Amidinotransferase catalyses a reversible transfer reaction of amidine groups from donor substrates to acceptors. It plays an important role in the formation of guanidine moiety of compounds in a wide range of organisms, from bacteria to higher plants and vertebrates 11, 12, 13, 14. For example, the l‐arginine:glycine amidinotransferase AGAT catalyzed the formation of guanidinoacetate in vertebrates creatine anabolism pathway, l‐arginine:glycine amidinotransferase CyrA involved in the biosynthesis of hepatotoxin cylindrospermopsin in *Cylindrospermopsis raciborskii*, and l‐arginine:inosamine phosphate amidinotransferase StrB1 involved in the biosynthesis of antibiotic streptomycin in *Streptomyces griseus* 11, 15, 16. Both AGAT and CyrA show identical enzymatic function involved in the formation of guanidinoacetic acid. Meanwhile, we learned that AGAT is more closely related to AmtA than CyrA based on phylogenetic analysis 16.

Human l‐arginine:glycine amidinotransferase (hAGAT) crystal structure and reaction mechanism have been well studied. The ligand‐induced structural changing of hAGAT looks like opening and closing of a lid, which was composed of the 300‐flap (Asp298Pro299Asn300Pro301Met302) and helix 9 15, 17. These residues confer the enzymes distinct functional characters. The AmtA's corresponding residues are Asp242Met243His244Pro245Met246 and CyrAs are Asp243Ile244Phe245Pro246Ser247. It was also found that in hAGAT, Asn300 and Met302 play important roles in substrate binding and stability 17, 18. To CyrA, substitution of Phe245 and/or Ser247 resulted in a broader substrate specificity 19.

The substrate specificity, both amidine donor and acceptor, of AmtA was examined by our research work and revealed in this article. Certain conserved amino acid residues involved in substrate binding were substituted and enzymatic activities of AmtA variants were examined as well.

## Materials and methods

### Protein overexpression and purification

The *amtA* gene was amplified by PCR with primer pair AmtAF (5′‐CAGCAAATGGGTCGCGGATCCATGCAACTCAATGAAAAAGATTCAA‐3′)/AmtAR (5′‐CGACGGAGCTCGAATTCGGATCCTCAAACCAAATAGCTTTCGAGTTTTCCT‐3′) from genomic DNA of *Pseudomonas syringae* pv. *phaseolicola* 1448A. Then it was inserted into the BamHI site of vector pET28a to give recombinant plasmid pWHU2007. Plasmid pWHU2007 was transferred into *Escherichia coli* BL21 and His‐tagged AmtA proteins were overexpressed and purified with standard protocols 20. AmtA M243P H244N and AmtA M246S variants encoding genes were synthesized by DetaiBio Co., Ltd (Nanjing, China) and were overexpressed using pET30a and purified with identical protocols. His‐tagged proteins were analyzed by SDS/PAGE and quantitated by a Nano‐Drop (Thermo, Waltham, MA, USA) instrument.

### Amidinotransferase enzyme assay

Substrates l‐Arg, l *‐h*Arg, l‐Orn, l‐Lys, l‐Gly, l‐Dap (l‐2,3‐Diaminopropionic acid), Guanidinoacetic acid, l‐Canavanine, Norleucine, and 6‐Aminocaproic acid were obtained from TCI Co., Ltd (Shanghai, China). The amidinotransferase enzyme assays were carried out in 1.5‐mL Eppendorf tubes with a system as 0.2 mg of purified protein, 20 mm Tris‐HCl (pH 8.0), 50 mm NaCl, 20 mm MgCl_2_, and 10 mm substrate, 100 μL in total. The reactions were performed at 37 °C for 1.5 h. AmtA (or substrates) was absent in the negative control reactions while the other factors remained identical. The reactions were terminated with equal volume of methanol and the precipitates were removed by centrifugation at 13 400 ***g*** for 10 min. The supernatants were lyophilized to dry out and then resuspended in 20 μL ddH_2_O for further derivatization.

### Precolumn derivatization of amino acids

The AccQ•Tag analysis kit was a product of Waters (Milford, MA, USA). Amino acid derivatization was conducted with AccQ•Tag reagents according to the manufacturer's protocol. Briefly, 10 μL of the amidinotransferase enzyme reaction solution was mixed with 70 μL of AccQ•Tag borate buffer and 20 μL of AccQ•Tag reagent solution and then incubated at 55 °C for 10 min.

### HPLC analysis

All HPLC analyses were carried out on a Shimadzu (Kyoto, Japan) HPLC instrument equipped with a degasser (DGU‐20A3), two pumps (LC‐20AT), an auto‐sampler (SIL‐20A), and a column oven (CTO‐20A). Chromatographic separation was achieved using an AccQ•Tag analysis column (3.9 mm × 150 mm, 4‐μm particles) at 37 °C. Reaction products were tested by monitoring absorption wave length at 254 nm by a PDA detector (SPD‐M20A).

## Results and Discussion

### AmtA catalyzed a reversible amidinotransferring reaction

**Figure 3:**
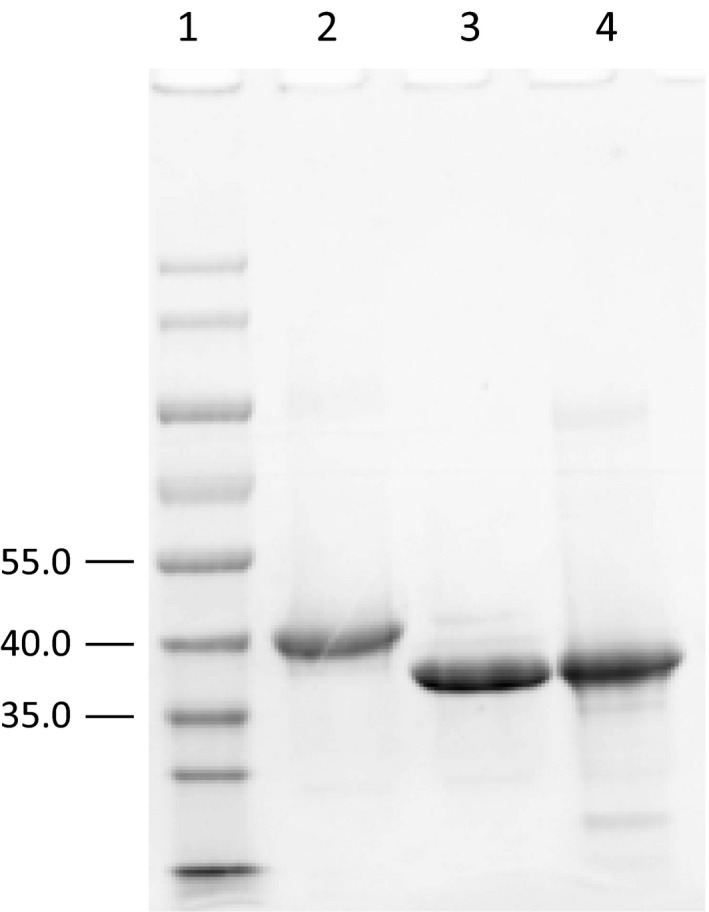
SDS/PAGE analysis of His‐tagged proteins. (1) Protein molecular weight standards (KDa); (2) His_(6)_·AmtA; (3) His_(6)_·AmtA M243P H244N; (4) His_(6)_·AmtA M246S.

**Figure 4:**
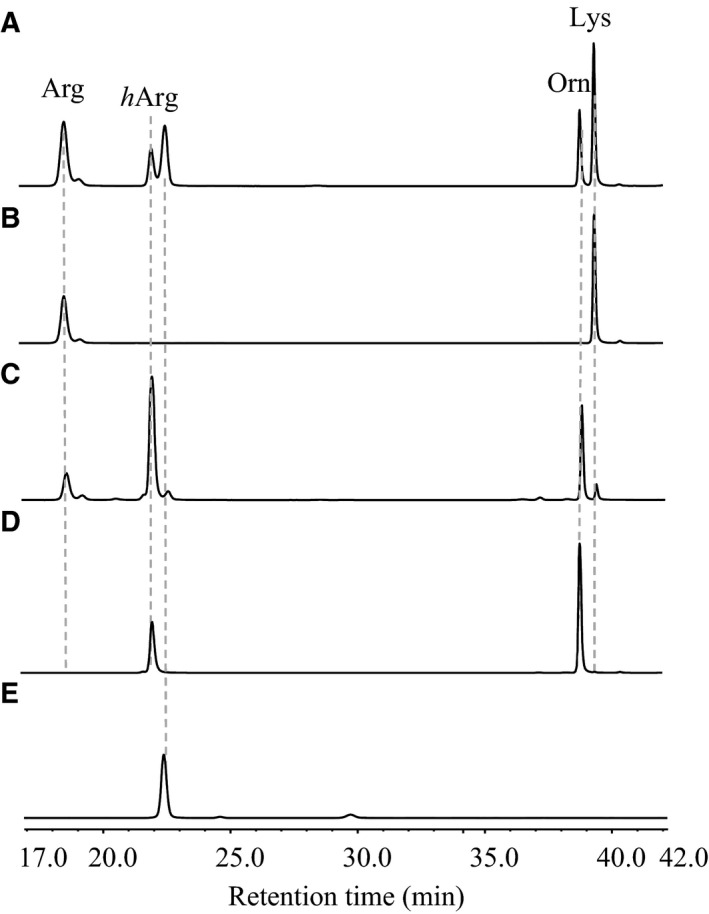
HPLC trace of AmtA‐catalyzed reversible reactions. (A) Forward reaction (with l‐Arg and l‐Lys as substrates); (B) negative control of forward reaction (enzyme AmtA was absent); (C) reverse reaction (with l‐*h*Arg and l‐Orn as substrates); (D) negative control of reverse reaction (enzyme AmtA was absent); (E) negative control (substrates were absent).

The SDS/PAGE results showed that the observed sizes of His_(6)_·AmtA, His_(6)_·AmtA M243P H244N and His_(6)_·AmtA M246S accord well with calculated molecular weights. His_(6)_·AmtA showed slight difference compared to the other two His‐tagged proteins because they were overexpressed using pET28a and pET30a respectively (Fig. 3). l‐Arg and l‐Lys were employed as amidine donor and acceptor respectively, in *h*Arg‐forming reactions catalyzed by AmtA. Anticipated reaction products, *h*Arg and l‐Orn were detected by HPLC analysis as shown in Fig. 4A. While, substrates l‐Arg and l‐Lys could not be converted to products completely even though the amount of AmtA enzyme was increased and the reaction time was extended dramatically. In the reverse reaction, *h*Arg and l‐Orn could be converted to l‐Arg and l‐Lys with limited conversion rate (Fig. 4C). It indicated that AmtA catalyzed a reversible amidinotransferring reaction. l‐Arg and l‐Lys are two proteinogenic amino acids, while *h*Arg and l‐Orn are biosynthetic precursors for phaseolotoxins (Fig. 1). Hence, AmtA could work as a regulating valve *in vivo* between primary metabolic pathway and the secondary metabolic pathway.

### l‐Canavanine was an alternative amidine donor for AmtA

**Figure 5:**
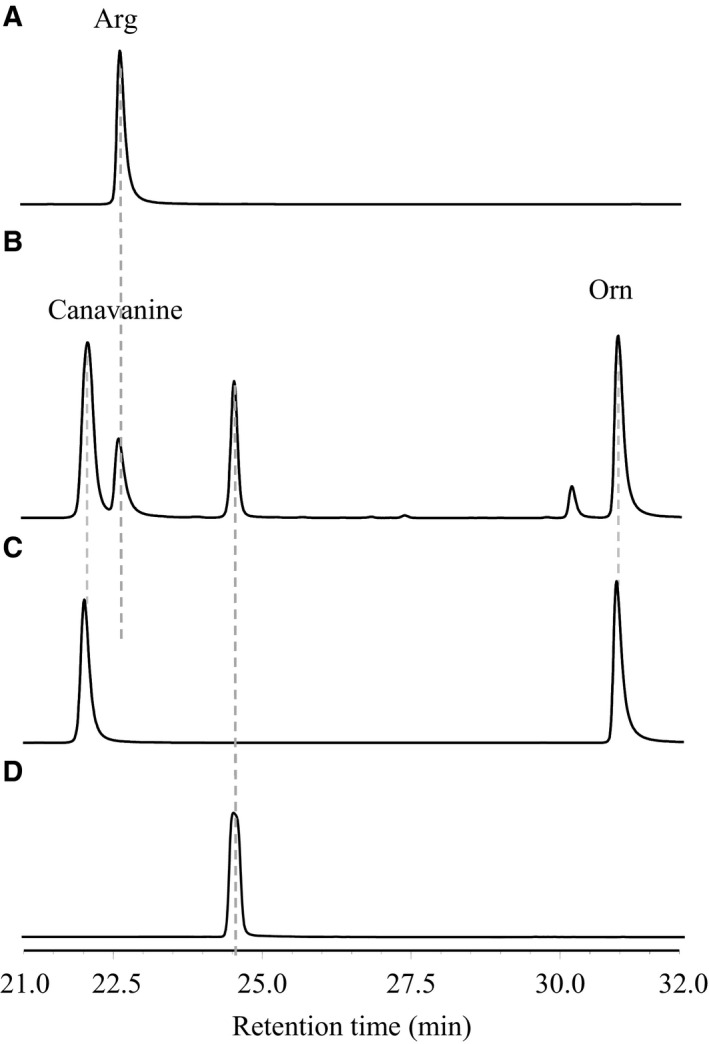
HPLC trace of AmtA‐catalyzed transamidination reaction with canavanine as amidine donor. (A) Authorized standard of l‐Arg, one of the targeted reaction products; (B) reaction with canavanine and l‐Orn as substrates; (C) negative control (enzyme AmtA was absent); (D) negative control (substrates were absent).

To test the substrate specificity of AmtA, l‐Canavanine and guanidinoacetic acid were employed as alternative amidine donors (as shown in Fig. 2B). l‐Orn performed as amidine acceptor in the reactions. Anticipated reaction product l‐Arg was detected only when l‐Canavanine was served as amidine donor (Fig. 5). This phenomenon may be attributed to the chemical structure similarity between arginine and canavanine. Guanidinoacetic acid is the natural amidine donor for hAGAT and CyrA, and an alternative amidine donor for StrB1 11, 15, 16. However, it was not accepted by AmtA. It showed that AmtA has stricter substrate specificity than these known amidinotransferases.

### Only l‐Lys and l‐Orn were acceptable amidine acceptors for AmtA

**Figure 6:**
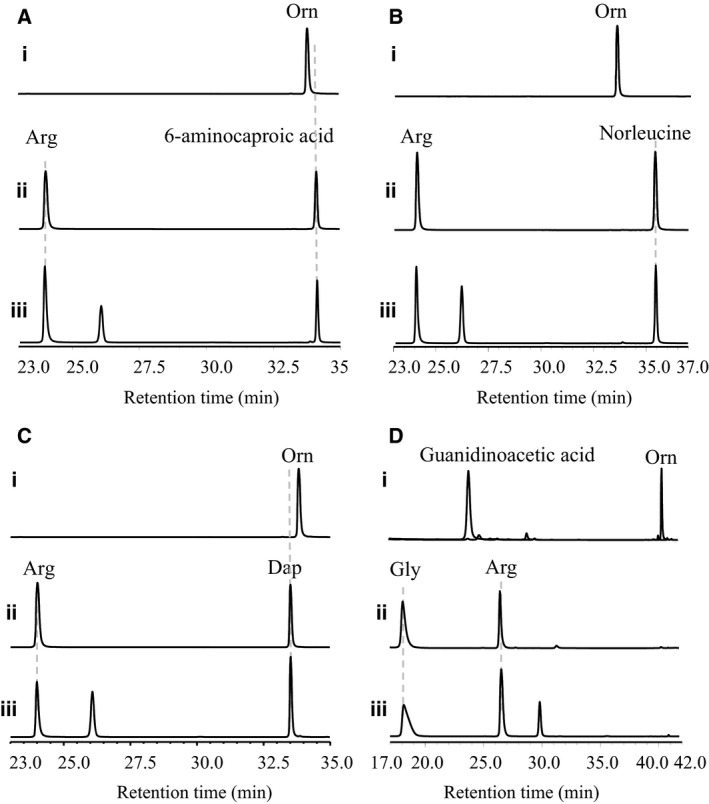
HPLC trace of AmtA‐catalyzed transamidination reactions with alternative amidine acceptors. (A) With l‐Arg as donor and 6‐Aminocaproic acid as acceptor; (B) with l‐Arg as donor and Norleucine as acceptor; (C) with l‐Arg as donor and l‐Dap as acceptor; (D) with l‐Arg as donor and l‐Gly as acceptor. (i) Authorized standards; (ii) negative control reactions (enzyme AmtA was absent); (iii) transamidination reactions.

Based on the aforementioned results, both l‐Lys and l‐Orn were acceptable amidine acceptors for AmtA. To check its substrate flexibility further, two groups of amidine acceptors were set in this research. The first group is Norleucine and 6‐Aminocaproic acid, which share identical carbon chain length with l‐Lys. However, they lack a C6 or C2 amino group, respectively. The second group is l‐Gly and l‐Dap, whose carbon chain length is shorter than Orn (Fig. 2C). In our research, none of these four molecules could be accepted as amidine acceptor when l‐Arg was served as amidine donor (Fig. 6). It indicates that both the carbon length and the two amino groups were essential factors in enzyme‐substrate recognition.

### AmtA M243P H244N variant showed identical substrate specificity with AmtA

**Figure 7:**
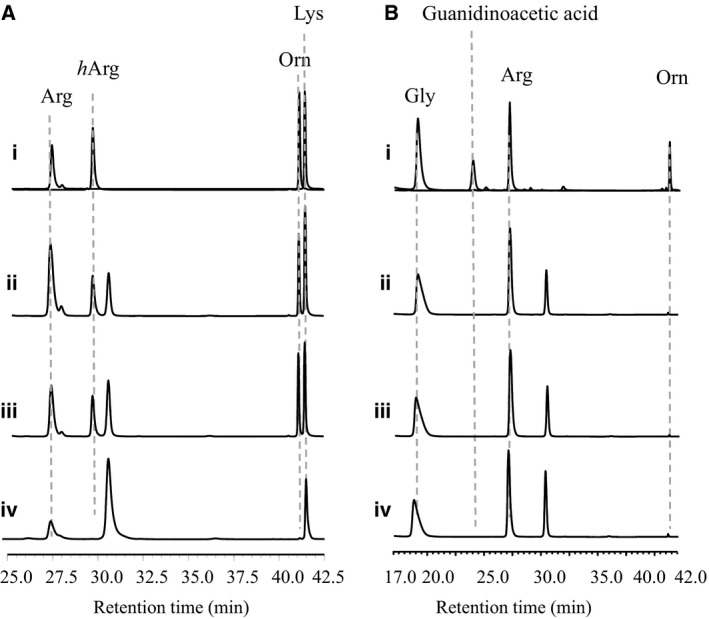
HPLC trace of AmtA and its variants catalyzed transamidination reactions. (A) With l‐Arg and l‐Lys as substrates; (B) with l‐Arg and l‐Gly as substrates. (i) Authorized standards; (ii) AmtA‐catalyzed reactions; (iii) AmtA M243P H244N‐catalyzed reactions; (iv) AmtA M246S‐catalyzed reactions.

AmtA is the nearest one to hAGAT in the phylogenetic tree among all prokaryotic amidinotransferase, including CyrA and StrB1 16. Glycine is an amidine acceptor for hAGAT and CyrA, but not for AmtA 15, 16, 17. In contrast to hAGAT, AmtA showed narrower substrate specificity. There are only two amino acid residue differences in the 300‐flap range between AmtA and hAGAT. To address the role of these two residues, AmtA M243P H244N variant was created and its substrate specificity was tested. *In vitro* reaction results showed that l‐Arg could serve as amidine donors and l‐Lys was acceptable amidine acceptors to AmtA M243P H244N variant (Fig. 7A‐iii). No detectable difference about catalytic efficiency between AmtA and AmtA variant was observed. l‐Gly was still not accepted by AmtA M243P H244N variant (Fig. 7B‐iii). To CyrA, substitution of residues in the active site with residues occurred at the corresponding positions of hAGAT (F245N and S247M) broadening its substrate specificity 19. In addition, l‐Lys is also an alternative amidine acceptor to wild‐type hAGAT 14. These cases indicate that the substrate specificity of the majority of amidinotransferases is flexible. Our results described here showed that AmtA is a unique member of amidinotransferases, whose substrate specificity is really stringent.

### AmtA M246S variant abolished amidinotransferase activity

The Met302 of hAGAT is essential for substrate binding, ligand‐induced structural changing and active center stability 15, 17. The AmtA's corresponding residue is Met246, while CyrA's is Ser247. So, the Met246 of AmtA was substituted by Ser, the amino acid occurred in CyrA. In our research, no detectable amidinotransferring activity was observed in the AmtA M246S variant (Fig. 7A‐iv,B‐iv). It showed that the Met246 of AmtA was a conserved amino acid as it was in hAGAT. Interestingly, we found that traceable amounts of Orn can be produced in the reaction (Fig. 7A‐iv,B‐iv). This phenomenon indicates that amidine donor, l‐Arg, could be hydrolyzed even under the condition that amidinotransferring activity was abolished. This observation accords well with the transamidination reaction mechanism of hAGAT 15. It seems that the amidine donors could be accepted correctly by AmtA M246S variant while the amidine acceptors could not. So, the amidine groups were removed from donors but could not be loaded on acceptors.

## Author contributions

CZ and ZD conceived and supervised the study; CZ designed experiments; ML and LC performed experiments; ML and CZ analyzed data; CZ and ML wrote the manuscript; CZ made manuscript revisions.
